# Gastric Trichobezoar Causing Gastrointestinal Bleeding: A Case Report

**DOI:** 10.7759/cureus.30282

**Published:** 2022-10-14

**Authors:** Fathia Harrabi, Houssem Ammar, Mahdi Ben Latifa, Rahul Gupta, Ali Ben Ali

**Affiliations:** 1 Gastrointestinal Surgery, Sahloul Hospital, University of Sousse, Sousse, TUN; 2 Gastrointestinal Surgery, Synergy Institute of Medical Sciences, Dehradun, IND

**Keywords:** melena, trichotillomania, trichophagia, hematemesis, trichobezoars

## Abstract

Trichobezoar is an underdiagnosed entity that has to be considered in children and adolescents, especially females, suffering from trichotillomania and trichophagia. Late diagnosis of trichobezoars showed that they cause gastrointestinal bleeding or perforation.

A 17-year-old girl patient was admitted with abdominal pain and gastrointestinal bleeding. On abdominal examination, a well-defined mass in the epigastrium and the left upper quadrant was identified. Upper gastrointestinal (GI) endoscopy identified an enormous trichobezoar, which was later removed by surgery.

Misdiagnosed gastric bezoars may cause life-threatening complications. Early detection for trichobezoar requires effective screening of trichotillomania. Psychiatric counseling is important to prevent bezoar recurrence.

## Introduction

Trichobezoar is defined as the accumulation in the stomach of non-absorbable human hair. It can extend through the pylorus to the duodenum, jejunum, and colon defining the Rapunzel syndrome [1]. It is a rare disorder with an incidence of less than 1% of the general population. On the other hand, 90% of trichobezoar cases in seen in young females with long hair aged between 13 and 20 years and suffering from trichotillomania (compulsion to pull hair) and trichophagia (compulsion to swallow hair) [2]. We report the case of a 17-year-old female with a large gastric trichobezoar presenting with upper gastrointestinal bleeding (melena, hematemesis).

## Case presentation

A 17-year-old girl presented with anemia and melena for four months. She also had hematemesis with abdominal discomfort, asthenia, and weight loss. She was a known case of iron deficiency anemia and was receiving treatment for the same.

**Figure 1:**
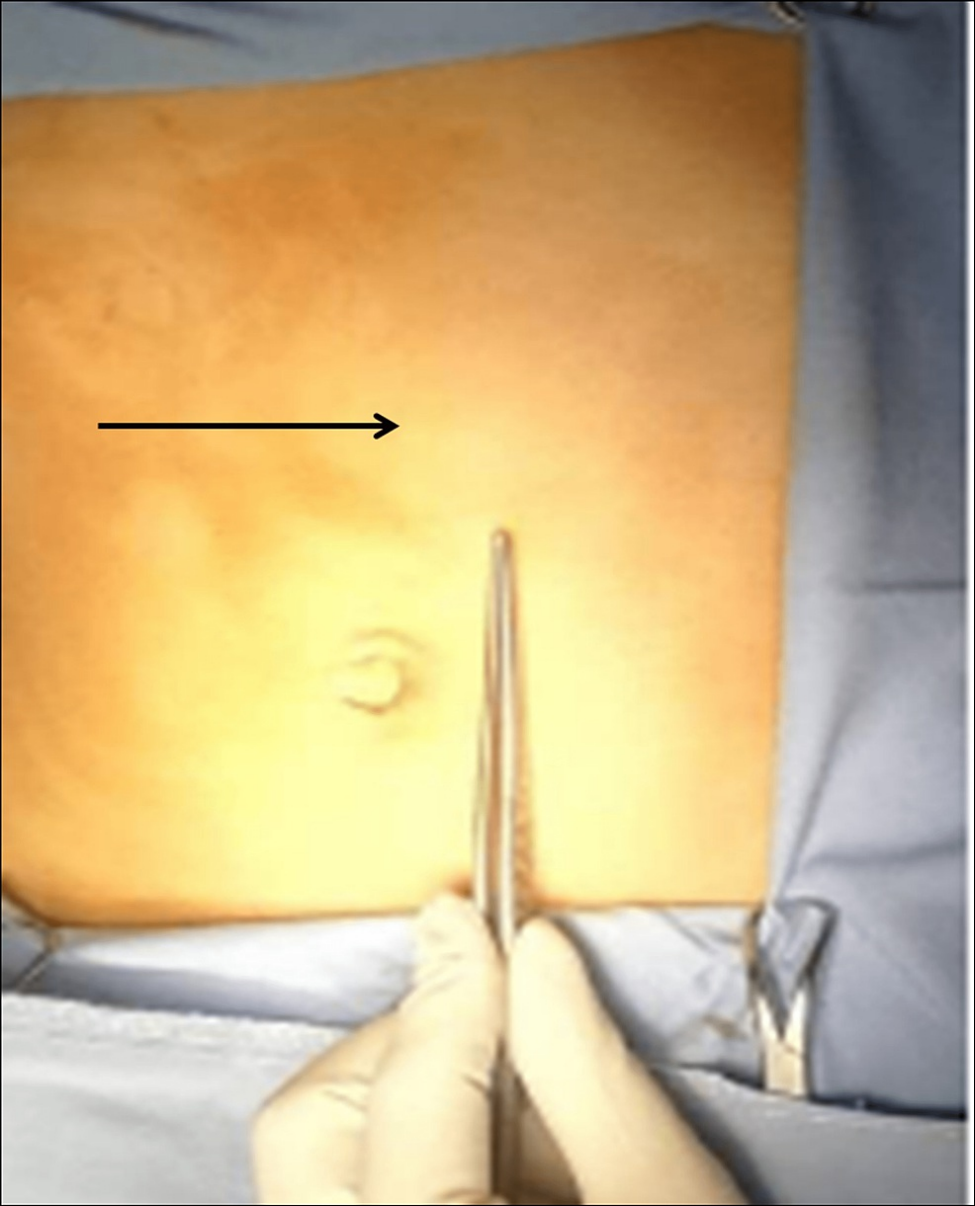
A firm mass in the epigastric region and the left upper quadrant (black arrow)

On physical examination the heart rate was 100 beats per minute, the blood pressure was 120\80 mmHg, and pulse oxygen saturation (SpO2) was 95%. Abdominal examination revealed an epigastric mass measuring around 8 × 6 cm, immobile, firm on palpation with smooth surface (Figure 1). Digital rectal examination confirmed melena. Laboratory investigations revealed a microcytic hypochromic anemia (hemoglobin (Hb): 8.3 gm/dl, mean corpuscular volume (MCV): 62 fL; mean corpuscular hemoglobin concentration (MCHC) < 310 g/l).

**Figure 2:**
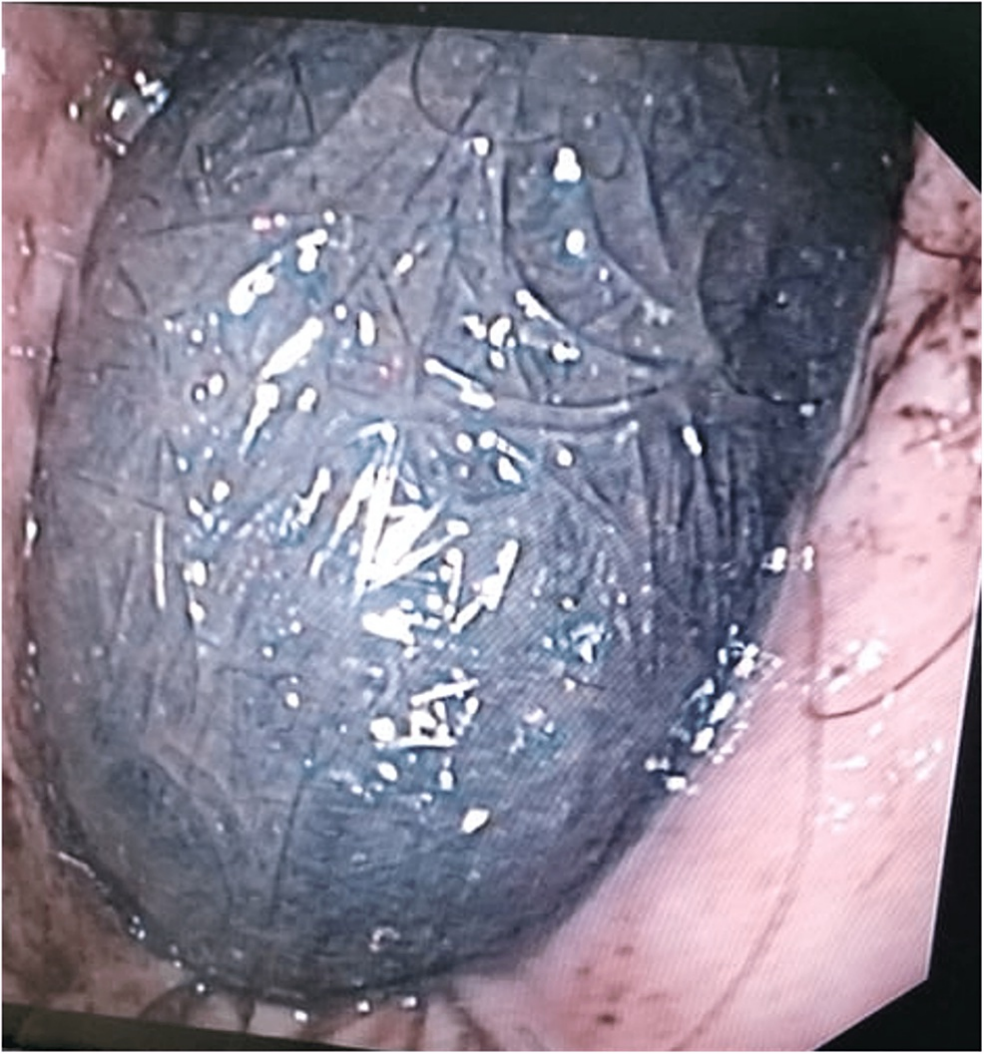
Upper gastrointestinal endoscopy showing a huge bezoar in the stomach with a petrous consistency

The patient underwent upper gastrointestinal endoscopy, which identified a huge bezoar in the stomach with a petrous consistency (Figure 2). There were bezoar-associated gastric ulcerations and bleeding. Abdominal computed tomography (CT) showed that the mass had completely filled the gastric cavity and extended into the duodenum.

**Figure 3:**
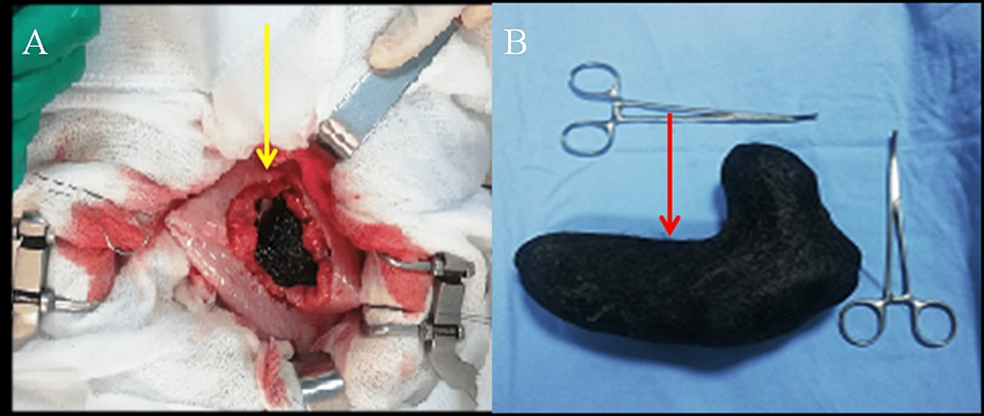
(A) Intraoperative images showing the trichobezoar after gastrotomy (yellow arrow); (B) The trichobezoar after its extraction (red arrow)

The patient and her family members were counseled regarding the available treatment options of endoscopic fragmentation and surgery. Due to the huge size of the bezoar, endoscopic fragmentation would have required multiple sessions and had the risk of failure. Hence, they opted for surgical removal of trichobezoar. The patient underwent surgical intervention using supraumbilical laparotomy, and anterior gastrotomy. A large trichobezoar with petrous consistency was removed from the stomach (Figure 3). 

The patient was discharged after five days of hospitalization. She underwent both psychiatric and psychological evaluations before discharge. Initially, she was ashamed of giving detailed history. But after recovering from the surgery she felt happy and gained the confidence to talk about her mental illness. She was diagnosed to have non-specific anxiety disorder and trichotillomania. She received anxiolytics and behavior therapy. At the last follow-up one year after her admission, the patient was symptom-free and receiving regular treatment from her psychologist.

## Discussion

The origin of the word “bezoar” is the Arabic word “bedzehr” and the Persian word “padzhar”. It is defined as the accumulation of non-absorbable human hair in the stomach [2]. About 90% of trichobezoars are found in female patients with long hair. The patient's medical history of coexistent psychiatric pathologic disorders with trichotillomania and trichophagia is important to suspect trichobezoar [1-3]. Trichotillomania, also called hair-pulling disorder, is a chronic mental disease, defined as the irresistible urge to pull out hair, despite trying to stop. It is more often observed in adolescents with a strong predominance in females and it is frequently associated with trichophagia, which is the compulsive eating of hair. When it is misdiagnosed, trichotillomania can cause serious complications such as low self-esteem, social setting problems, skin hair damage, and trichobezoar. In our case, the mental illness was not diagnosed until she developed gastric trichobezoar. When undiagnosed in their early stages, gastric bezoars may cause gastric ulceration, perforation, hemorrhage, and obstruction [2,4]. 

The symptoms of gastric trichobezoar are usually nonspecific, including abdominal pain nausea, vomiting, digestive bleeding, iron-deficiency anemia, epigastric discomfort, early satiety, indigestion, and weight loss [2,5]. This diagnostic delay is due to a lack of communication by the patients with trichotillomania, who feel ashamed of their disease and try to hide it. It can also be due to the lack of experience of general physicians to detect these illnesses. Upper gastrointestinal endoscopy is the modality of choice to confirm the diagnosis. It can also be used for therapeutic purposes in early cases to avoid complications [6].

Currently, minimally invasive procedures such as endoscopic fragmentation and removal or laparoscopic extraction of trichobezoar are the preferred choices for treatment [5-7]. However, the literature provides no evidence of the superiority of these techniques over open surgery. Only a few cases of endoscopic removal of trichobezoar after fragmentation have been reported in the English literature. However, the endoscopic removal of all fragments requires multiple sessions and has a risk of pressure ulceration and, in rare cases, esophageal perforation. Also, satellite fragments of a large trichobezoar might migrate through the pylorus after fragmentation, causing intestinal obstruction and the removal of those fragments is impossible through endoscopy [7-9]. That’s why surgical extraction is still considered the treatment of choice for trichobezoar. Surgical extraction can be performed by open or laparoscopic approach. The laparoscopic approach has the advantages of faster recovery, small incisions, and better cosmesis [6,8,9]. However, laparoscopic surgery may not be suitable in all cases. In our case, the bezoar was huge and with petrous consistency with a high risk of failure of endoscopic treatment. Moreover, we did not have the required expertise to perform laparoscopic removal of trichobezoar. Hence, open surgical extraction was performed. Lastly, in addition to surgical treatment, psychiatric consultation is crucial to prevent relapses and treat comorbid conditions that are often associated with this disease.

## Conclusions

Trichobezoar is an underdiagnosed entity that has to be considered in children and adolescents, especially females, suffering from trichotillomania and trichophagia. The early detection of these diseases can prevent gastrointestinal complications such as bleeding or perforation. Psychiatric counseling plays an important role to prevent bezoar recurrence.
